# Machine Learning Estimates of Natural Product Conformational Energies

**DOI:** 10.1371/journal.pcbi.1003400

**Published:** 2014-01-16

**Authors:** Matthias Rupp, Matthias R. Bauer, Rainer Wilcken, Andreas Lange, Michael Reutlinger, Frank M. Boeckler, Gisbert Schneider

**Affiliations:** 1 Department of Chemistry and Applied Biosciences, Eidgenössische Technische Hochschule (ETH), Zürich, Switzerland; 2 Department of Pharmaceutical Chemistry, Eberhard Karls University, Tübingen, Germany; University of Maryland, Baltimore, United States of America

## Abstract

Machine learning has been used for estimation of potential energy surfaces to speed up molecular dynamics simulations of small systems. We demonstrate that this approach is feasible for significantly larger, structurally complex molecules, taking the natural product Archazolid A, a potent inhibitor of vacuolar-type ATPase, from the myxobacterium *Archangium gephyra* as an example. Our model estimates energies of new conformations by exploiting information from previous calculations via Gaussian process regression. Predictive variance is used to assess whether a conformation is in the interpolation region, allowing a controlled trade-off between prediction accuracy and computational speed-up. For energies of relaxed conformations at the density functional level of theory (implicit solvent, DFT/BLYP-disp3/def2-TZVP), mean absolute errors of less than 1 kcal/mol were achieved. The study demonstrates that predictive machine learning models can be developed for structurally complex, pharmaceutically relevant compounds, potentially enabling considerable speed-ups in simulations of larger molecular structures.

## Author Summary

Molecular dynamics simulations provide insight into the dynamic behavior of molecules, e.g., into the adopted spatial arrangements of its atoms over time. Methods differ in the approximations they employ, resulting in a trade-off between accuracy and speed that ranges from highly accurate but expensive quantum mechanical calculations to fast but more inaccurate molecular mechanics force fields. Machine learning, a sub-discipline of artificial intelligence, provides algorithms that learn from data, that is, make predictions based on previously seen examples. By starting with a few expensive quantum mechanical calculations, training a machine learning algorithm on them, and then using the resulting model to carry out the molecular dynamics simulation, one can improve the accuracy/speed trade-off. We have developed and applied such a hybrid quantum mechanics/machine learning approach to Archazolid A, a natural product from the myxobacterium *Archangium gephyra* and a potent inhibitor of vacuolar-type ATPase. By dynamically refining our model over the course of the simulation, we achieve errors of less than 1 kcal/mol while saving over 40% of the quantum mechanical calculations. Our study demonstrates the feasibility of predictive machine learning models for the dynamics of structurally complex, pharmaceutically relevant compounds, potentially enabling considerable speed-ups in simulations of even larger biomolecular structures.

## Introduction

Molecular dynamics (MD) simulations allow for computing low-energy molecular conformations, which is essential when rapid heuristic or empirical approaches fail or are deemed too coarse-grained [1], [2]. MD simulations can be performed at different levels of sophistication, ranging from empirical and semi-empirical methods to quantum mechanical (QM) approaches. The computational power required increases with higher levels of theory, rendering exact energy estimation of large or complex chemical structures practically limited. Here we show that fast machine learning (ML) methods may serve as surrogate energy estimators for computationally demanding MD studies of structurally complex natural products, taking as an example Archazolid A, a macrolide from the myxobacterium *Archangium gephyra*.

In first principles MD simulations, electronic structure calculations are repeatedly carried out for highly similar conformations of the same molecule. Information from previous calculations is usually ignored. As an exception, ML algorithms have been used to exploit this information by interpolating between reference calculations, yielding fast (ms instead of hours), accurate, highly empirical energy estimates [3]. For the interpolation of potential energy surfaces in molecular dynamics, this approach has been limited to small systems due to the molecular representation used. Here, we provide proof of principle that such “QM/ML” approaches can also be developed for structurally complex, pharmaceutically relevant compounds, yielding highly accurate predictions.

### Target

**Figure 1:**
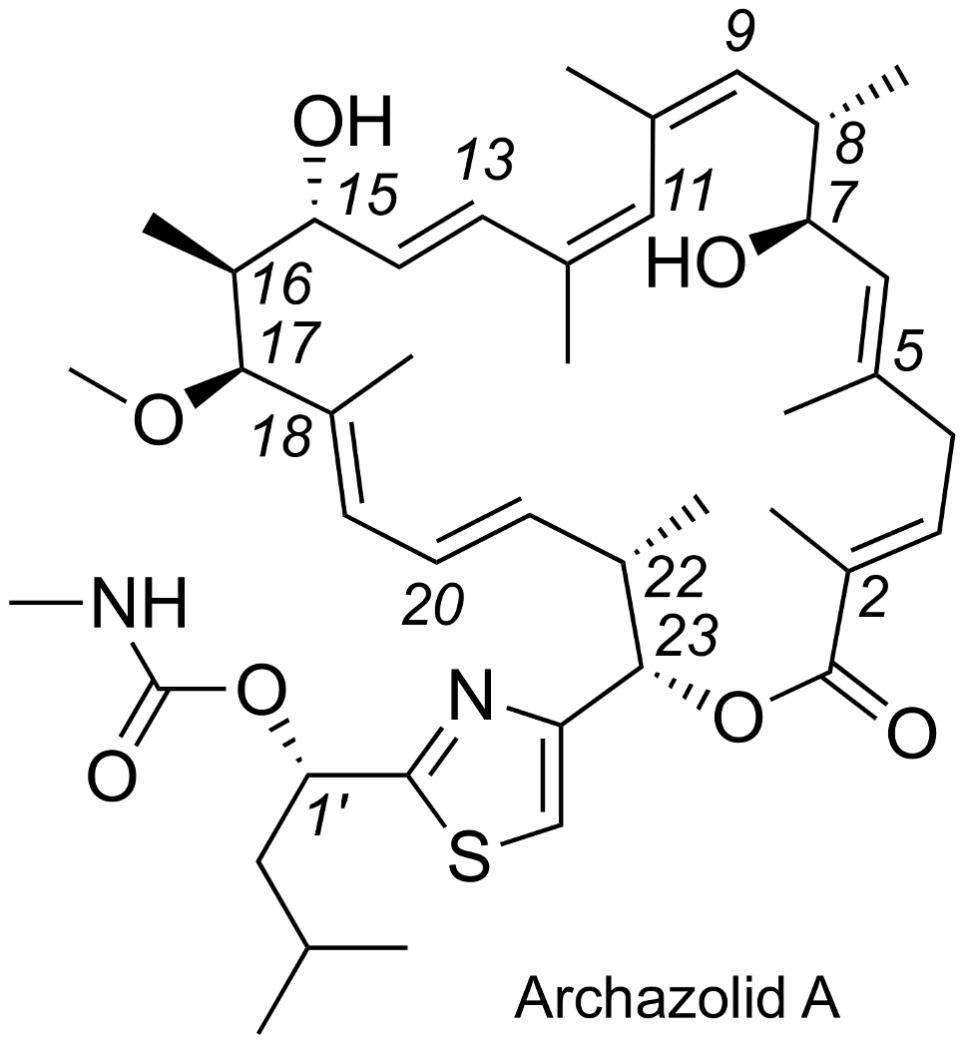
Configuration of the myxobacterial polyketide Archazolid A, a potent inhibitor of vacuolar-type ATPase (V-ATPase).

**Figure 2:**
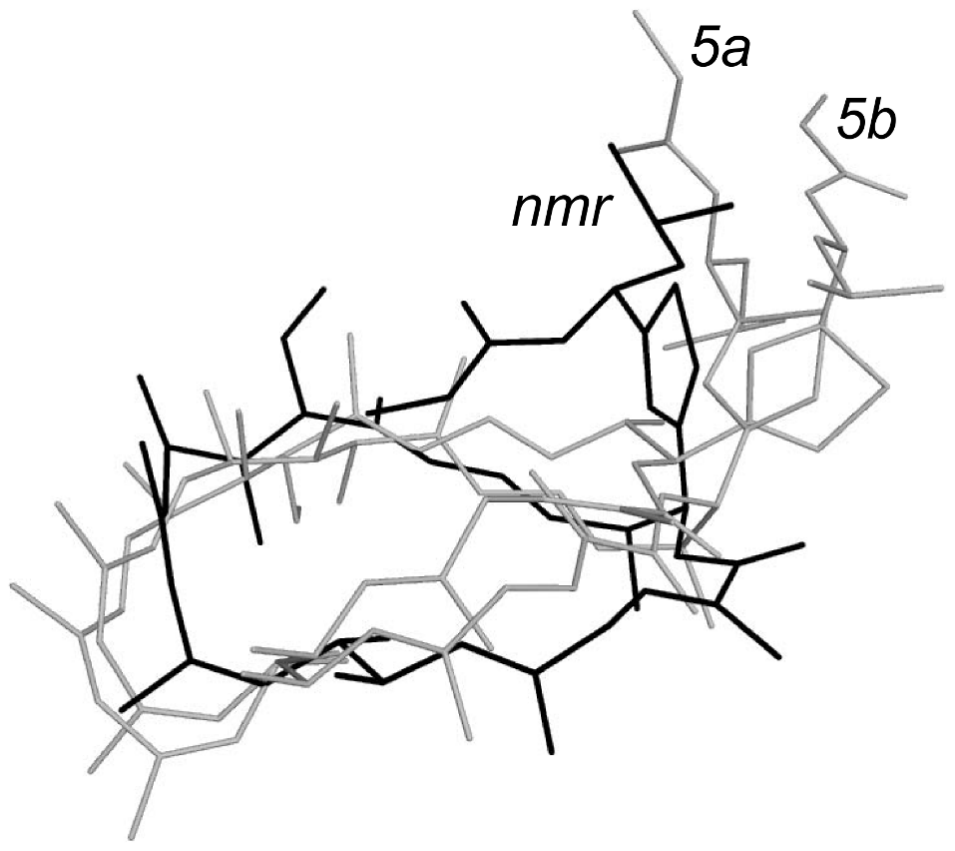
Reported conformations *5a*, *5b* (grey), and *nmr* (black) of Archazolid A derived from NMR studies [9]. Molecules were superimposed by minimizing root mean square deviation in PyMol (www.pymol.org).

Archazolid A (molecular weight of 739 Da; Fig. 1) is a low-nanomolar inhibitor of vacuolar-type ATPase (V-ATPase) with anti-proliferative activity *in vitro* and *in vivo* [4]–[8]. Its central 24-membered macrolactone ring contains seven alkenes, and eight methyl- and hydroxyl-bearing stereocenters. Their full relative and absolute stereochemistry (2E, 5E, 7S, 8S, 9Z, 11Z, 13E, 15R, 16S, 17S, 18E, 20E, 22S, 23S, 1′S) and three in-solution model conformations were elucidated by Menche and coworkers using nuclear magnetic resonance (NMR) spectroscopic methods (Fig. 2) [9].

### Machine learning

The common idea behind ML models for QM is that whenever a series of computationally expensive, related QM calculations is done, ML can be used to replace some of these calculations to speed up the process. The way ML does this is by interpolating between a set of reference calculations, the training set, with the underlying assumption being that similar chemical systems have similar properties (the “chemical similarity principle” [10]). This approach is well known in cheminformatics, where experimentally determined molecular properties are estimated [11]. ML has recently been used in diverse QM contexts ranging from density functional theory [12] over prediction of atomization energies across chemical compound space [13] to transition state theory [14].

ML estimators of potential energy surfaces have been pursued since the early 1990s using artificial neural networks [3], [15]–[17]. Recently, non-parametric methods such as Gaussian process regression have been used as well [18]–[23] (a parametric model absorbs all information from the training data into its parameters, e.g., the weights of an artificial neural network. A non-parametric model requires access to the training data. Another way to view this is that parametric methods use a fixed number of basis functions, whereas non-parametric methods use one basis function per member of the training set. Thus, for non-parametric methods the complexity of the model can increase with the number of training data. Note that non-parametric models can have parameters). A critical component of QM/ML models is the representation of the simulated system, i.e., the choice of molecular descriptor. Neural networks have often been used with symmetry functions [3], which have advantages with respect to periodic potentials, but do not scale to larger systems. Other representations include internal coordinates, system-specific variables, and more complex procedures. For example, Gaussian approximation potentials [22] use local atom densities, project them onto the four-dimensional unit sphere, calculate (hyper)spherical harmonics coefficients, and finally use their bispectrum, a three-point correlation function, to obtain a fixed-length set of cubic rotational invariants [23]. In contrast, we use a simple matrix representation based on nuclear charges and intra-molecular distances only [13].

## Results

### Molecular dynamics

Starting from a modelled Archazolid A conformation using the NMR constraints published by Farès *et al.* [9], we generated an ensemble of conformations using semi-empirical MD at the AM1 level [24] in VAMP [25]. Four different trajectories of 300 ps length were generated at a temperature of 400 K, ensuring an enhanced sampling rate. Similar conditions were previously successfully applied to the rational design of 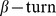 inducing peptide mimetics [26], [27].

MD snapshots were first relaxed with AM1 in MOPAC [28]. Full optimization of each structure was then achieved with TURBOMOLE [29], applying a BLYP(RI)-D2-COSMO/def2-SVP (DFT-D2) level of theory. Assessing the relative conformational energy differences of the DFT-D2 optimized snapshots, using BLYP(RI)-D3-COSMO/def2-TZVP (DFT-D3), revealed a broad distribution of energies spanning 88 kJ/mol (Fig. S1). With progression of the MD trajectories, the energies of the resulting snapshots fluctuated around an average value, except for trajectory 2, where progressively worse conformers were generated after 100 ps. A number of conformers obtained in all four trajectories were found to be energetically favored at the same level as the published NMR-motivated structures *nmr*, *5a*, *5b* [9]. More than 50% of the DFT-D2 optimized snapshots possessed relative energies below that of conformation *5a* (<35 kJ/mol), and about 20% of the generated conformers possessed a more favorable energy than conformer 5b (<20 kJ/mol). 20 conformers were at a similar energy level as *nmr* (<8 kJ/mol).

Overall, the conformers generated comply with between 13 and 25 of the experimental ROESY (rotating-frame nuclear Overhauser effect) constraints, while structures reported in the literature [9] satisfy between 18 and 23 constraints. Structures obtained from the first 100 ps of each trajectory seem to deviate more strongly from the average number of satisfied NMR constraints than structures toward the end of the trajectories (Fig. S1).

In summary, the semi-empirical MD sampling and DFT-D2 optimization produced conformers possessing favorable energies and similar compliance with experimental data as the previously reported conformations *5a*, *5b*, and *nmr*. The MD study suggested prominent flexibility of Archazolid A. While most of the conjugated double bonds were found to be co-planar in the minimized structures, the 1,3,5-hexatrien moiety between atoms 9 and 16 did not show full co-planarity, but most often torsion angles of 50–60° between the double bonds in positions 9 and 11 (Fig. S2), which is in agreement with both the NMR-derived conformations *5a*, *5b*, and models of the Archazolid-V-ATPase complex [30], [31]. The importance of this region for bioactivity and bioavailability is supported by preliminary structure-activity relationship data available for Archazolid analogs, which highlight the importance of the C-7 hydroxyl as part of the pharmacophore [4], [32]. We thus concluded that our MD simulations sampled relevant conformations of the central macrocyclic structure.

To compare the conformational space from our QM/DFT-D methodology with that of force fields (FF), we generated 2 800 diverse conformers using the MMFF94x FF. We then clustered both FF-based and QM-based conformations with respect to geometric measurements of the macrolactone ring. About 40% of the FF-based structures are in clusters containing no QM-based conformers. Likewise, about 40% of QM-based conformations are found in clusters containing hardly any FF-based structures (<10%). In the mixed clusters, no correlation was found between normalised relative energies of FF-based and QM-based conformations. On account of this, only DFT-D energies were considered for further study.

**Figure 3:**
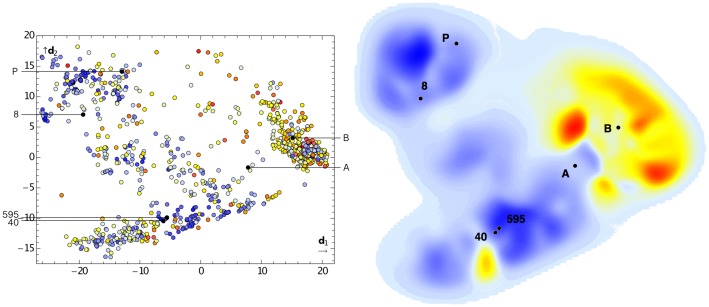
Projection of MD conformations of Archazolid A onto two dimensions (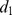, 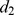) by principal component analysis. Shown are distribution of individual conformations (left) and smoothed energy landscape generated by LiSARD [52] (right). Labels indicate reported NMR-motivated structures (A = *c5a*, B = *c5b*, P = *nmr*) and lowest-energy MD conformations (8, 595, 40). Color coding is from lowest (blue) to highest (red) relative energy.

We visualized the computed DFT energy landscape by projecting the conformations sampled by the four MD simulations using principal component analysis (PCA, Fig. 3). PCA is a dimensionality reduction method that preserves global distances (see Figs. S3, S4, S5, S6 for visualization using stochastic neighbor embedding, a technique that preserves local distances). In the two-dimensional projection, we observed adjacent, potentially connected low-energy basins (blue regions in Fig. 3), which also contained conformation *nmr*. Conformer *d008*, located close to *nmr*, is the lowest-energy structure from all MD runs.

### Machine learning

We trained ML models to capture the relationship between the relaxed Archazolid A conformations sampled from the MD simulation and their DFT-D3 energies.

Conformations were encoded using a simple matrix representation [13] based solely on nuclear charges *Z* and inter-atomic distances *D*, the same input that enters first principles calculations. In brief, off-diagonal elements of the symmetric matrix were computed as 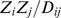, where 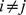 are atom indices, and main diagonal elements as 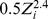. This representation is related to atom-pair and distance-scaled molecular autocorrelation descriptors [33], [34]. Due to symmetry and fixed composition and geometry, only the strict lower triangular part of the matrix was used, concatenated into a 6441-dimensional vector. Note that due to strong correlation between descriptors, the effective dimensionality is much lower (90% (95%, 99%) of the variance in the descriptors is explained by the first 25 (46, 136) PCA components. Relevant dimension analysis [35], a related technique taking energies into account, estimates the dimensionality to be 89).

Gaussian processes [36], sometimes known as Kriging, are a non-parametric regression method with regularization to prevent over-fitting. GP models take the form
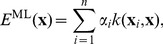
(1)where 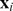 is the *i*-th reference conformation, *n* is the number of reference conformations, **x** is a new conformation to be predicted, 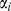 are regression coefficients, and *k* is a kernel function. Kernels, also called covariance functions, are symmetric positive definite functions that measure the similarity between data points, here conformations in the vector representation described above. We used the linear kernel 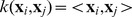. For each prediction, GPs also provide the predictive variance, a built-in measure of the domain of applicability that can be used to quantify confidence into individual predictions.

### Validation results

**Figure 4:**
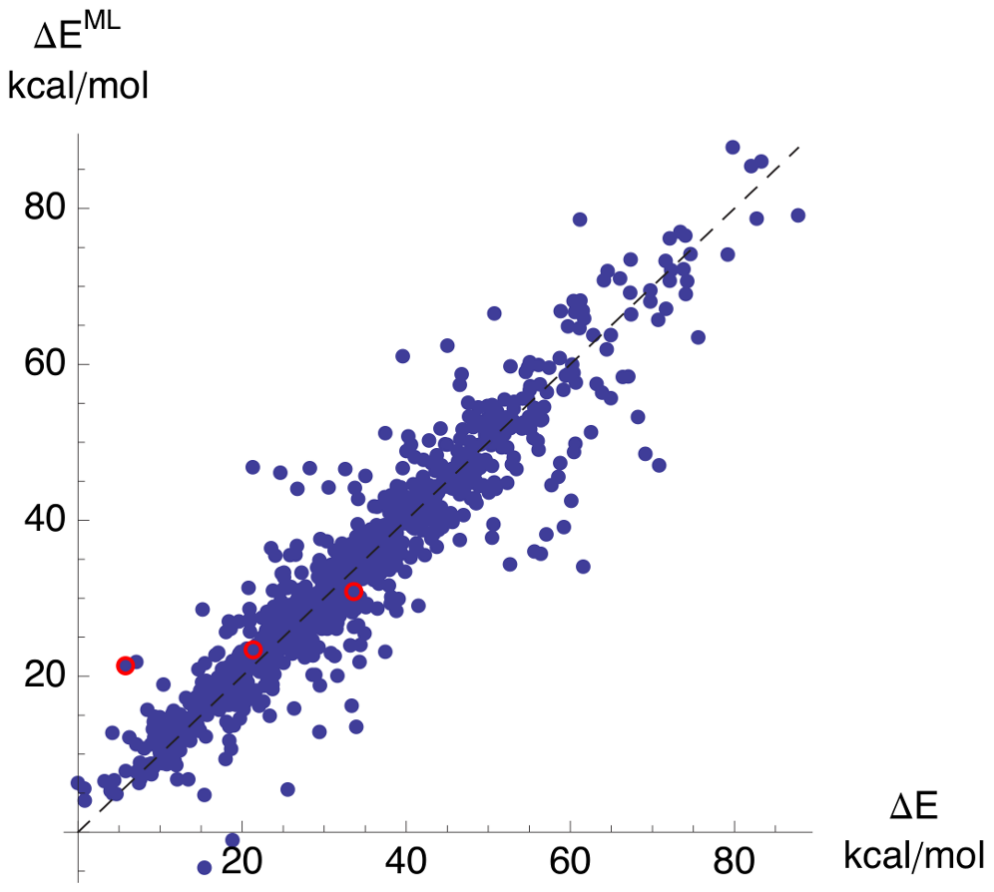
Predicted 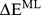 vs calculated ΔE values of Archazolid A conformations. All predictions were obtained by stratified 10-fold cross-validation of the complete MD data. NMR-based conformations *c5a*, *c5b*, *nmr* are marked by red circles (external test data).

Retrospective validation of predictive accuracy on all MD data using 10 repetitions of 10-fold stratified cross-validation (*n* = 100) yielded a root mean squared error (RMSE) of 5.35±0.72 kJ/mol, mean absolute error (MAE) of 3.51±0.38 kJ/mol, and squared correlation coefficent of *R*^2^ = 0.88±0.03 (see Fig. 4 for a scatterplot).

For cross-validation, data were divided into 10 parts (splits) of equal size and similar distribution of energy (stratification by energy). For each split *i*, a ML model was trained on the other splits 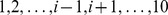 and used to predict split *i*. This provides predictions for all conformations by models trained on 90% of the data, never including the predicted conformation itself. Model parameters were optimized in an inner loop of cross-validation (nested cross-validation). See refs. [37], [38] for more detailed explanations.

The large number of 6441 descriptors introduces the possibility of chance correlations [39], [40] between descriptors and energies. Although this risk is lessened by correlations between descriptors (resulting in fewer actual degrees of freedom) and our use of regularization, we performed two randomization tests (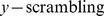) [41] with permuted labels and descriptors, respectively. This resulted in *p*-values of <10^−35^ (Mann-Whitney U-test, *n* = 100) and an increase in estimated noise levels of three and five orders of magnitude, respectively, strongly indicating that the observed good performance of our model is genuine.

**Figure 5:**
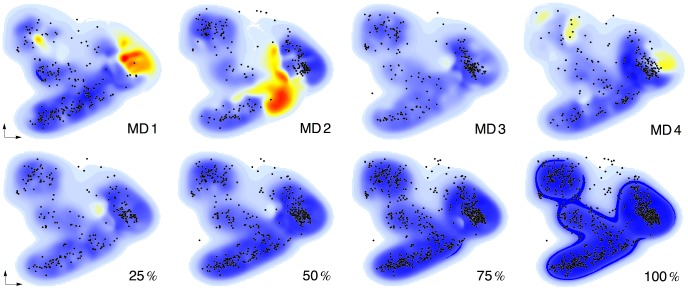
Influence of sampling. Shown are smoothed PCA maps of absolute prediction errors for ML models trained on individual MD data (top row) and ML models trained on randomized subsets of all MD data (bottom row). Color indicates magnitude of error (blue = low, red = high); training samples are shown as black dots.

The importance of sampling is well known in MD, and has led to the development of various sampling schemes [1], such as umbrella sampling [42] or reconnaissance metadynamics [43]. Similarly, sampling also affects ML models via the sampling of reference conformations. We demonstrate this as follows: First, we trained ML models using all conformations from one of the MD runs as training data (Table 1). Training data were almost perfectly replicated (*R*^2^>0.99), but markedly lower predictive performance on the other MD runs revealed imperfect conformational sampling of each MD simulation alone. Then, we combined all data from the four MD runs, and trained ML models using random subsets of 25, 50, 75 and 100% of the computed Archazolid A conformations (Table 2). This resulted in clearly improved predictions on test data, i.e., conformations that were not contained in the training set. Fig. 5 shows the relationship between sampling (data density) and prediction errors.

**Table 1:** Performance of ML models trained separately on each individual MD run and tested on the other MD runs.

	MD run 1		MD run 2		MD run 3		MD run 4	
	train	test	train	test	train	test	train	test
*n*	237	726	238	725	244	719	244	719
RMSE	0.00	15.46	1.08	12.12	3.50	8.29	0.26	11.31
MAE	0.00	10.21	0.82	8.92	2.61	6.19	0.20	8.00
MAE (%)	0.00	11.73	1.03	10.16	3.00	7.43	0.28	9.11
*R*^2^	1.00	0.50	1.00	0.49	0.93	0.73	1.00	0.62

RMSE: root mean square error (kJ/mol), MAE: mean absolute error (kJ/mol), MAE (%): MAE as a percentage of the range of training set energy values, *R*^2^: squared Pearson correlation coefficient.

**Table 2:** Performance of ML models trained on randomized subsets of increasing size of the complete MD data.

	25%		50%		75%		100%	
	train	test	train	test	train	test	train	test
*n*	240	723	481	482	722	241	963	0
RMSE	3.32	7.59	2.09	6.21	1.93	5.52	1.04	–
MAE	2.56	5.54	1.55	4.36	1.45	3.48	0.72	–
MAE (%)	3.10	6.31	1.88	4.97	1.67	4.21	0.82	–
*R*^2^	0.95	0.76	0.98	0.84	0.98	0.87	1.00	–

See Table 1 for abbreviations.

The simplest way to use a ML model is to create a large amount of training data, then train and apply the model. As shown (Tables 1 and 2), it is important that the training data are diverse because only conformations covered by them will be predicted well; the larger such a training set is, the better the predictions. In an MD simulation, such a training set could be obtained by a fixed-size initial sampling at elevated temperature. This corresponds roughly to the situation in Table 2.

**Figure 6:**
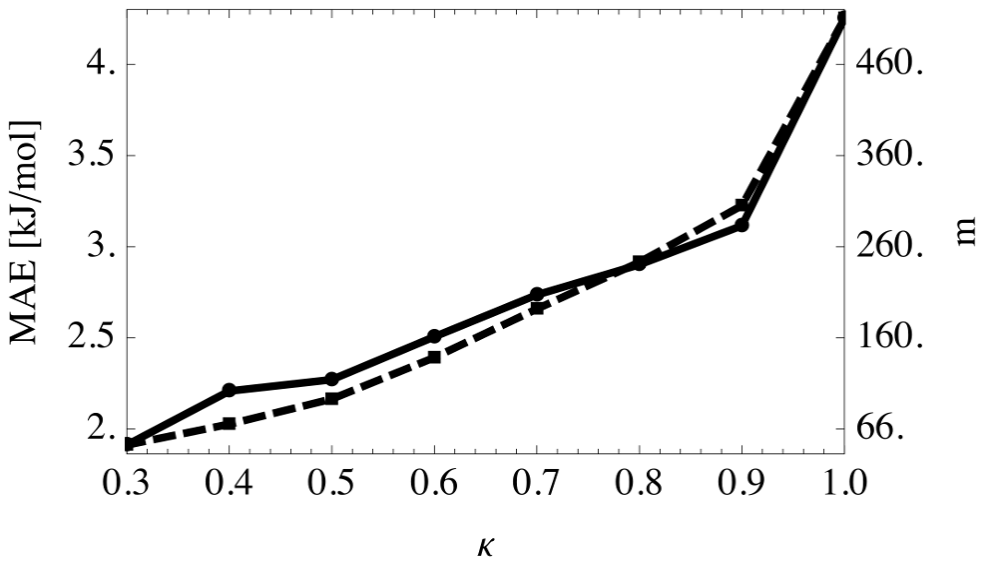
Learning using predictive variance. Shown is the trade-off between mean absolute error (MAE, solid line, left scale) and number of predicted conformations (*m*, dashed line, right scale). Results are averaged over all possible orderings of the four MD runs (4! = 24; standard deviations ca. 0.4 kJ/mol and 35 samples). Squared correlation is *R*^2^ = 0.99.

A more economical way to use a ML model is to adjust the model on the fly [44]: Start with a small initial training set. Then, for each new conformation, decide whether the model can predict it. If not predicted, add it to the training set and retrain the model. This adaptive scheme requires a measure of the domain of applicability [45], [46] of the model. Here, we use the GPs predictive variance: If it is below the *κ* = 0.95 quantile of the predictive variance of the training data, the conformation is accepted for prediction. Note that *κ* can be used to trade off prediction accuracy versus computational savings, i.e., the number of predicted conformations (Fig. 6). Using an initial stratified training set of 50 conformations and *κ* = 0.95 yielded a RMSE of 4.42±0.41 kJ/mol, MAE of 3.46±0.34 kJ/mol, and squared correlation of *R*^2^ = 0.94±0.01 for 384±42 predicted conformations (mean ± std. dev., averaged over all 4! = 24 orderings of the four MD runs). For this study, this would have saved 23 out of 58 days used for DFT-D3 calculations (single core).

## Discussion

This study demonstrates that GP regression, a Bayesian non-parametric ML method, is suited for modeling relationships between molecular structure and QM properties even for structurally complex, pharmaceutically relevant compounds. The simple molecular representation used proved sensitive to structural variations in relaxed geometries and enabled finding correlations between simulated conformations and computed energy values at the DFT/BLYP-disp3/def2-TZVP level of theory. For Archazolid A, mean absolute errors of less than 4 kJ/mol (≈1 kcal/mol) were achieved. The GP's predictive variance was used to dynamically improve the model over the course of the MD simulation (“learning on the fly”) [44].

Here, we did not use the ML model's derivatives. For this, note that due to the highly empirical nature of these models, derivatives can only be expected to be accurate along directions covered by the training data. To avoid excessive generation of training data, projected gradients can be employed [12]. Note that the model is readily usable for Monte Carlo simulations.

Adaptive sampling strategies for MD like reconnaissance metadynamics [43] bias the course of the MD simulation based on the trajectory so far by avoiding low-energy regions that have already been sampled sufficiently. Since the training set of an effective non-parametric ML model by necessity covers the conformational space visited so far, it provides a natural means to bias the MD simulation. For GP models, the predictive variance, which is effectively a measure of training data density, could be used. Active learning [47] might be useful for such sampling strategies as well.

Innovative ML algorithms that are tightly integrated with MD techniques could provide access to long-term simulations of challenging chemical and biomolecular systems. Here, we made a successful first step in this direction taking myxobacterial Archazolid A as an example.

## Materials and Methods

### Molecular dynamics simulation

Semi-empirical MD simulations were carried out with VAMP using the AM1 Hamiltonian [24], [25]. A starting structure of Archazolid A was modeled using NMR constraints published by Farès *et al.* [9]. This model was then minimized with constraints [9] using the MMFF94x force-field in MOE (Molecular Operating Environment, 2011.2010; Chemical Computing Group, Montreal, Canada) and further refined to closely match the conformation of the NMR-derived Archazolid A structure. Before starting the MD simulation, the model structure was minimized in VAMP using the AM1 Hamiltonian. Four trajectories of 300 ps length at a temperature of 400 K were calculated using an NVT ensemble with a Berendsen heat bath coupling constant of 40 fs. For solving Newtons equations of motion, the velocity Verlet integrator within VAMP was used and initial velocities of particles were set according to the Maxwell distribution. The total linear momentum of the system was forced to zero to prevent drifting. Using a time step of 1 fs for the molecular dynamics simulations, snapshots were recorded every 100 fs resulting in trajectories of 3000 snapshots. The initial 50 ps of each trajectory were discarded to ensure equilibration of the system. A total of 1000 equally distributed conformers (1 ps distance) from the four trajectories were energy minimized using MOPAC2012 at AM1 level (Stewart Computational Chemistry, Colorado Springs, USA). The geometry optimization was conducted with a molecular mechanics correction to amide bonds, a dielectric constant of 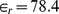 for the COSMO simulation and precise settings. Optimization failed for 21 conformers, leaving 979 structures.

### DFT calculations

Subsequently, we performed DFT-D2 calculations using TURBOMOLE (v6.3.1, TURBOMOLE GmbH, Karlsruhe, Germany) [29] to further optimize the MD snapshots. Geometry optimizations were performed at the BLYP(RI)-D2-COSMO/def2-SVP level with the dielectric constant set to 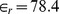. We obtained a total of 963 DFT-D2 optimized snapshots. To allow for direct comparison of MD snapshots and the experimentally determined NMR structures, we additionally conducted DFT-D2 optimizations for these structures. Final energies were obtained using single point calculations on the BLYP(RI)-D3-COSMO/def2-TZVP level with the dielectric constant set to 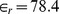 and using the third-generation Grimme dispersion correction [48], [49]. Calculations were done on a cluster with Intel Xeon E5440 (2.83 GHz, 800 MB RAM/core) processors (DFT-D2 optimization) and on a cluster with AMD FX-8150 (3.6 GHz, 800 MB RAM/core) processors (DFT-D3 single point energies).

### Assessment of minimized MD snapshots

The 1H-NMR ROESY correlations published by Hassfeld *et al.* [30] were used to assess the agreement of in silico generated conformers with experimentally determined constraints of Archazolid A. The ROESY correlations were classified into the following 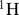 distance constraints: <5 Å for weak, <3.5 Å for medium and <2.5 Å for strong ROESY correlations [50]. Proton-proton and proton-methyl distances were calculated in MOE. For proton-methyl correlations, the average distance to all methyl protons was calculated to yield an average distance. Computed distances were then assessed using the NMR-derived ROESY constraints yielding the number of satisfied distance constraints for each conformer. Constraints that were either always or never fulfilled by all conformations were omitted in the analysis.

### Comparison to force fields

The MMFF94x FF was used to carry out low mode MD simulations in MOE. We accepted only conformations within an energy window of ΔE = 20.0 kcal/mol of the found global minimum and treated conformations within an RMSD of 0.25 after energy minimization and optimal rigid body superposition as identical. Conformational sampling was terminated when 100 consecutive attempts failed to generate any novel conformation, yielding about 2 800 structures.

### Principal component analysis (PCA)

PCA [51] finds uncorrelated directions of maximum variance in the data. These are given by the eigenvectors of the empirical covariance matrix (sorted in descending order of the corresponding eigenvalues, which also provide a measure of the explained variance). The number of principal components to use is a free parameter of the method. Here, we used two components for visualization. PCA projections were done using Mathematica (version 9, Wolfram Research).

### Smoothed energy landscapes

Two-dimensional representations of the data colored by DFT-D3 values provide detailed information about relationships between conformations. To enable better identification of global features like energy basins and barriers, we smooth these energy landscapes as described elsewhere [52]. In brief, the Nadaraya-Watson estimator [53], [54] with Gaussian kernel was used to obtain locally weighted averages at locations without observations. The involved bandwidth was estimated using the normal reference rule [55], resulting in local density adaptive bandwidths. Smoothed energy landscapes were calculated using the visualization software LiSARD (version 1.2.2, ETH Zürich, Switzerland; for license requests, contact G. Schneider). The smoothing factor was set to *k* = 0.3.

### Gaussian process regression

Gaussian process (GP) regression is a Bayesian non-parametric technique [36], [56], [57]. A GP is a generalization of the normal distribution to functions, i.e., a function-valued random variable. For regression, one considers all functions generated by a GP that “agree” with the training data, i.e., one conditions a joint Gaussian prior distribution on it. The mean of the resulting posterior distribution is the predictor; its variance can be used as a measure of confidence in the prediction (domain of applicability). In matrix notation, predictor and predictive variance take the form
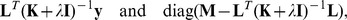
where **K**, **L**, **M** are the kernel matrices between training conformations, training and test conformations, and test conformations, respectively, *λ*>0 is a hyper-parameter controlling regularization strength, **I** is the identity matrix, and **y** is the vector of reference energies. The regression coefficients in Eq. 1 are thus given by 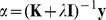. Note that GP predictions are technically equivalent to those of kernel ridge regression [58], a regularized form of ordinary regression. A GP is specified by a covariance function, or kernel, that quantifies similarity between two inputs. We used the linear kernel 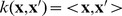. Models with the non-linear squared exponential kernel did not lead to significant improvements in performance (Table S2). The noise level hyper-parameter *λ* (the variance of the assumed label noise) was chosen by optimizing the stratified 10-fold cross-validated mean absolute error over a logarithmic grid, For performance estimates, this was done in an inner loop of cross-validation.

## Supporting Information

Figure S1 **Assessment of minimized molecular dynamics snapshots.** (a) Fulfilled ROESY constraints versus trajectory sequence. Optimized structures are color-coded as green filled diamonds (published NMR-motivated conformations), yellow filled circles (trajectory 1), red squares (trajectory 2), purple diamonds (trajectory 3), and blue triangles (trajectory 4). Trend lines are shown using the same color-coding. (b) Shown are the three previously published structures (A, B, and C; see also main text Fig. 2), and five structures generated by the simulations (D–H). These conformers exhibit favorable relative energies or a high number of fulfilled ROESY constraints. (c) Relative DFT-D3 energies versus satisfied ROESY constraints. (d) Relative DFT-D3 energies versus trajectory sequence.
(PDF)

Figure S2 **Computed low energy conformations *d8*, *d239*, *d595* of Archazolid A.** The conformers display torsion angles close to 55° between the double bonds in positions 9 and 11 (arrows).
(PDF)

Figure S3 **Smoothed principal components analysis visualizations.** Shown are projections to the first two principal components smoothed by Lisard using conformations relaxed by AM1 (a,c) and DFT-D2 (b,d), colored by DFT-D2 (a,b) and DFT-D3 energies (c,d).
(PDF)

Figure S4 **Smoothed stochastic neighbor embedding visualizations.** Shown are two-dimensional embeddings smoothed by Lisard using conformations relaxed by AM1 (a,c) and DFT-D2 (b,d), colored by DFT-D2 (a,b) and DFT-D3 energies (c,d).
(PDF)

Figure S5 **Smoothed principal components analysis visualizations with minimum energy conformations.** Shown are projections to the first two principal components smoothed by Lisard using conformations relaxed by AM1 (a,c) and DFT-D2 (b,d), colored by DFT-D2 (a,b) and DFT-D3 energies (c,d).
(PDF)

Figure S6 **Smoothed stochastic neighbor embedding visualizations with minimum energy conformations.** Shown are two-dimensional embeddings smoothed by Lisard using conformations relaxed by AM1 (a,c) and DFT-D2 (b,d), colored by DFT-D2 (a,b) and DFT-D3 energies (c,d).
(PDF)

Table S1 **Lowest energy conformations.** Shown are, for all four scenarios, the three MD conformations with lowest relative energy and the three NMR-motivated conformations. ident. = identifier, ind. = index (1-based), ΔE = relative energy.
(PDF)

Table S2 **Performance of machine learning models.** Statistics are over 10 runs of 10-fold stratified cross-validation (*n* = 100). For each entry, mean ± standard deviation are shown. The same splits are used in each row. All preprocessing (centering, standardization) is done separately for each split, on training folds data only. Optimization of hyper-parameters (noise level, length scale) is done in an inner loop of stratified 10-fold cross-validation using a logarithmic grid. All units are in kJ/mol. In all scenarios, machine learning models significantly outperform the null model. Standardization and/or centering never improve performance by more than one standard deviation. Investigated machine learning models: Model names have form *abc*, with *a* indicating the kernel (0 = linear, 1 = squared exponential), *b* indicating standardization (0 = no, 1 = yes), and *c* indicating centering in kernel space (0 = no, 1 = yes). Note that the 011 model is redundant as standardization centers the input vectors.
(PDF)
