# Supplementary material for: Machine Learning Estimates of Natural Product Conformational Energies
Source: PLoS Comput Biol. 2014 Jan 16;10(1):e1003400. doi: 10.1371/journal.pcbi.1003400 (PMC3894151; doi:10.1371/journal.pcbi.1003400)
Supplement: Figure S1 — Assessment of minimized molecular dynamics snapshots. (a) Fulfilled ROESY constraints versus trajectory sequence. Optimized structures are color-coded as green filled diamonds (published NMR-motivated conformations), yellow filled circles (trajectory 1), red squares (trajectory 2), purple diamonds (trajectory 3), and blue triangles (trajectory 4). Trend lines are shown using the same color-coding. (b) Shown are the three previously published structures (A, B, and C; see also main text Fig. 2), and five structures generated by the simulations (D–H). These conformers exhibit favorable relative energies or a high number of fulfilled ROESY constraints. (c) Relative DFT-D3 energies versus satisfied ROESY constraints. (d) Relative DFT-D3 energies versus trajectory sequence. (PDF) [file pcbi.1003400.s001.pdf]

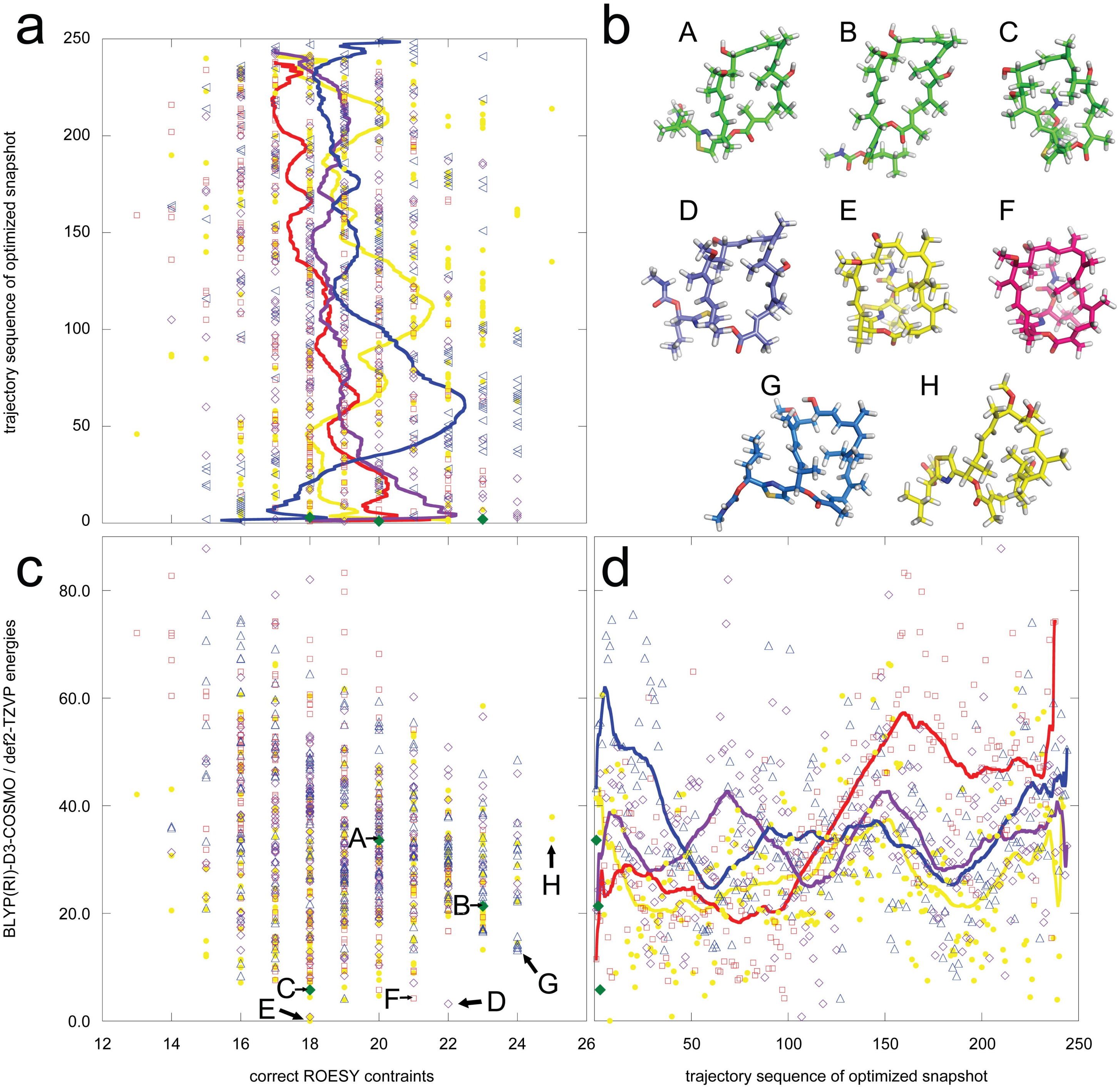

Figure S1: **Assessment of minimized molecular dynamics snapshots.** (a) Fulfilled ROESY constraints versus trajectory sequence. Optimized structures are color-coded as green filled diamonds (published NMR-motivated conformations), yellow filled circles (trajectory 1), red squares (trajectory 2), purple diamonds (trajectory 3), and blue triangles (trajectory 4). Trend lines are shown using the same color-coding. (b) Shown are the three previously published structures (A, B, and C; see also main text Fig. 2), and five structures generated by the simulations (D-H). These conformers exhibit favorable relative energies or a high number of fulfilled ROESY constraints. (c) Relative DFT-D3 energies versus satisfied ROESY constraints. (d) Relative DFT-D3 energies versus trajectory sequence.
