# Supplementary material for: Machine Learning Estimates of Natural Product Conformational Energies
Source: PLoS Comput Biol. 2014 Jan 16;10(1):e1003400. doi: 10.1371/journal.pcbi.1003400 (PMC3894151; doi:10.1371/journal.pcbi.1003400)
Supplement: Figure S2 — Computed low energy conformations d8, d239, d595 of Archazolid A. The conformers display torsion angles close to 55° between the double bonds in positions 9 and 11 (arrows). (PDF) [file pcbi.1003400.s002.pdf]

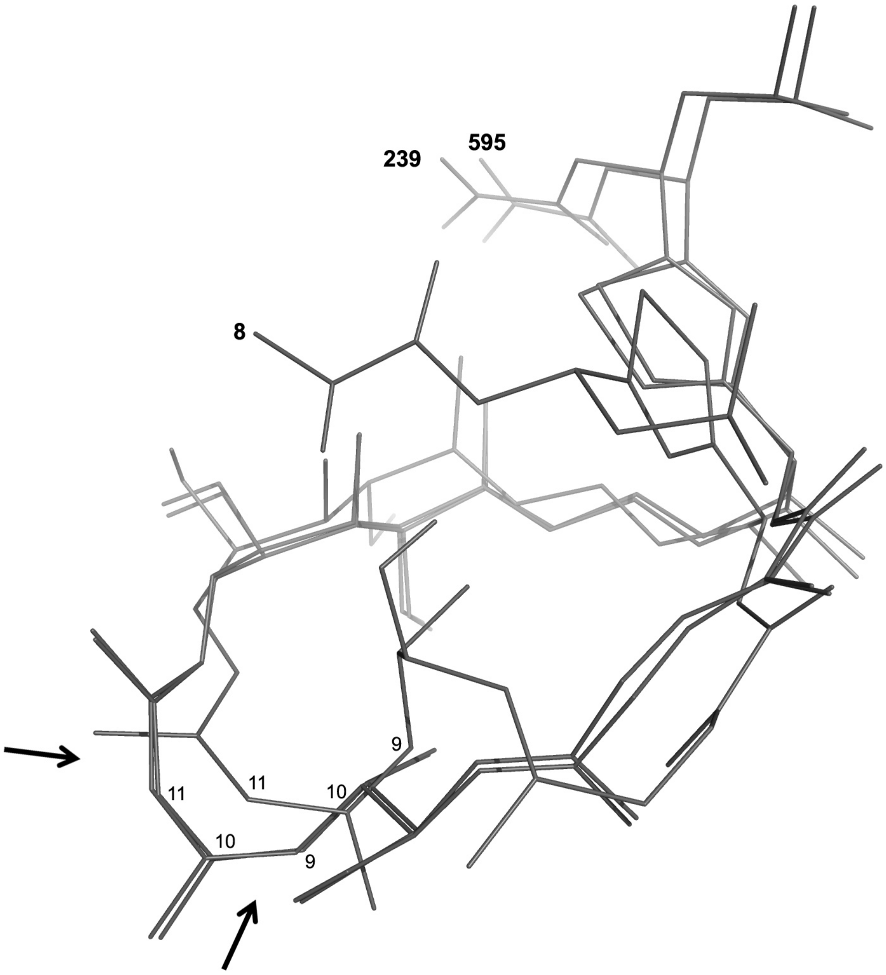

Figure S2: **Computed low energy conformations *d8*, *d239*, *d595* of Archazolid A.** The conformers display torsion angles close to 55 ° between the double bonds in positions 9 and 11 (arrows).
