# Supplementary material for: Machine Learning Estimates of Natural Product Conformational Energies
Source: PLoS Comput Biol. 2014 Jan 16;10(1):e1003400. doi: 10.1371/journal.pcbi.1003400 (PMC3894151; doi:10.1371/journal.pcbi.1003400)
Supplement: Figure S5 — Smoothed principal components analysis visualizations with minimum energy conformations. Shown are projections to the first two principal components smoothed by Lisard using conformations relaxed by AM1 (a,c) and DFT-D2 (b,d), colored by DFT-D2 (a,b) and DFT-D3 energies (c,d). (PDF) [file pcbi.1003400.s005.pdf]

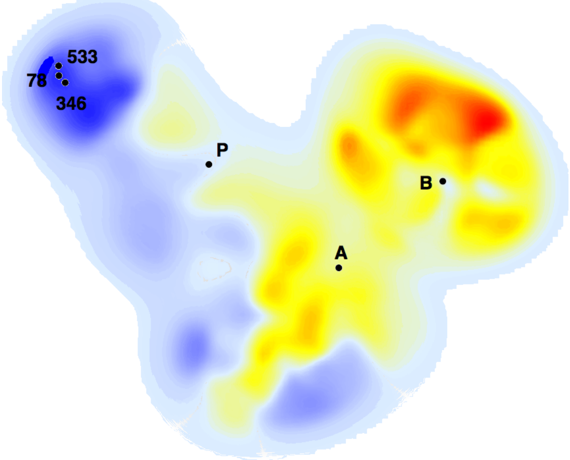

(a) AM1 conformations colored by DFT-D2 energies

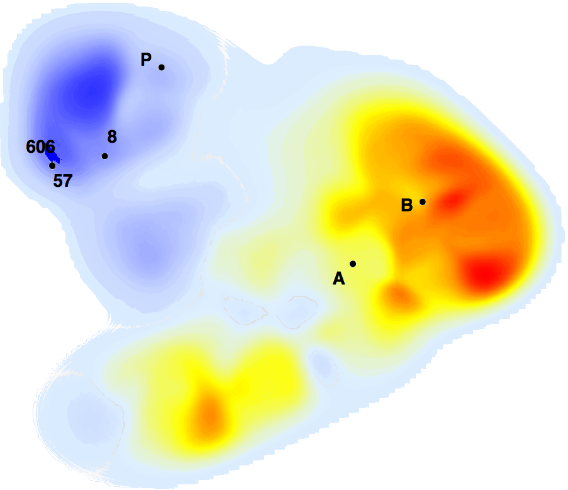

(b) DFT-D2 conformations colored by DFT-D2 energies

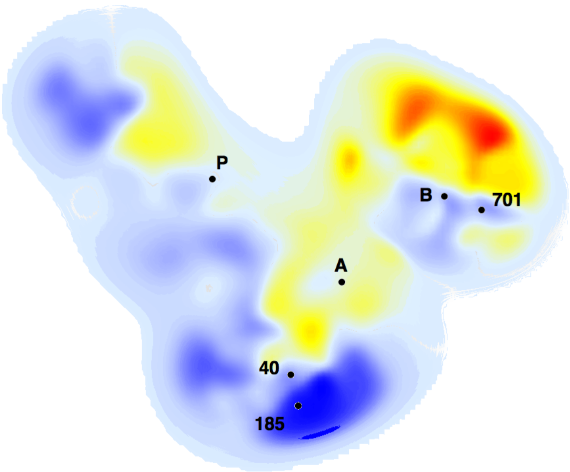

(c) AM1 conformations colored by DFT-D3 energies

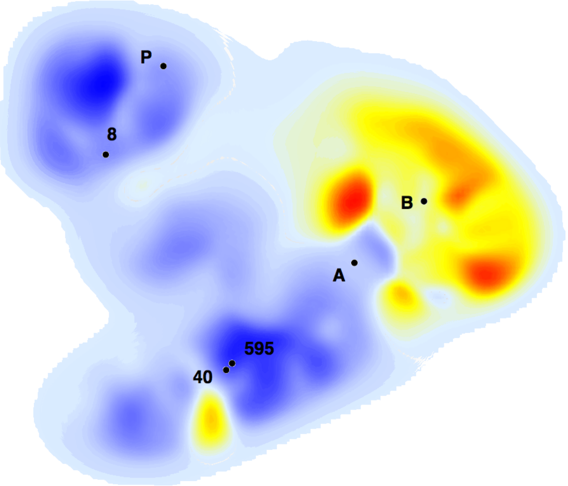

(d) DFT-D2 conformations colored by DFT-D3 energies

Figure S5: **Smoothed principal components analysis visualizations with minimum energy conformations.** Shown are projections to the first two principal components smoothed by Lisard using conformations relaxed by AM1 (a,c) and DFT-D2 (b,d), colored by DFT-D2 (a,b) and DFT-D3 energies (c,d).
