# Supplementary material for: Machine Learning Estimates of Natural Product Conformational Energies
Source: PLoS Comput Biol. 2014 Jan 16;10(1):e1003400. doi: 10.1371/journal.pcbi.1003400 (PMC3894151; doi:10.1371/journal.pcbi.1003400)
Supplement: Table S1 — Lowest energy conformations. Shown are, for all four scenarios, the three MD conformations with lowest relative energy and the three NMR-motivated conformations. ident. = identifier, ind. = index (1-based), ΔE = relative energy. (PDF) [file pcbi.1003400.s007.pdf]

Table S1: **Lowest energy conformations.** Shown are, for all four scenarios, the three MD conformations with lowest relative energy and the three NMR-motivated conformations. ident. = identifier, ind. = index (1-based), ΔE = relative energy.

(a) AM1/DFT-D2

ident.	rank	ind.	ΔE
anmr	1	3	0.0
a078	2	78	21.8
a346	3	338	24.6
a533	4	524	29.7
ac5b	12	2	37.3
ac5a	24	1	42.8

(b) AM1/DFT-D3

ident.	rank	ind.	ΔE
a701	1	691	0.0
a040	2	43	0.1
a185	3	183	2.3
anmr	4	3	3.4
ac5a	61	1	14.3
ac5b	62	2	14.3

(c) DFT-D2/DFT-D2

ident.	rank	ind.	ΔE
d008	1	11	0.0
d057	2	57	5.5
d606	3	596	5.7
dnmr	68	3	30.3
dc5b	512	2	64.9
dc5a	628	1	71.2

(d) DFT-D2/DFT-D3

ident.	rank	ind.	ΔE
d008	1	11	0.0
d595	2	585	0.8
d040	3	43	0.8
dnmr	11	3	5.8
dc5b	227	2	21.3
dc5a	535	1	33.6
