# Supplementary material for: Machine Learning Estimates of Natural Product Conformational Energies
Source: PLoS Comput Biol. 2014 Jan 16;10(1):e1003400. doi: 10.1371/journal.pcbi.1003400 (PMC3894151; doi:10.1371/journal.pcbi.1003400)
Supplement: Table S2 — Performance of machine learning models. Statistics are over 10 runs of 10-fold stratified cross-validation (n = 100). For each entry, mean ± standard deviation are shown. The same splits are used in each row. All preprocessing (centering, standardization) is done separately for each split, on training folds data only. Optimization of hyper-parameters (noise level, length scale) is done in an inner loop of stratified 10-fold cross-validation using a logarithmic grid. All units are in kJ/mol. In all scenarios, machine learning models significantly outperform the null model. Standardization and/or centering never improve performance by more than one standard deviation. Investigated machine learning models: Model names have form abc, with a indicating the kernel (0 = linear, 1 = squared exponential), b indicating standardization (0 = no, 1 = yes), and c indicating centering in kernel space (0 = no, 1 = yes). Note that the 011 model is redundant as standardization centers the input vectors. (PDF) [file pcbi.1003400.s008.pdf]

Table S2: **Performance of machine learning models.** Statistics are over 10 runs of 10-fold stratified cross-validation ($n = 100$). For each entry, mean ± standard deviation are shown. The same splits are used in each row. All preprocessing (centering, standardization) is done separately for each split, on training folds data only. Optimization of hyper-parameters (noise level, length scale) is done in an inner loop of stratified 10-fold cross-validation using a logarithmic grid. All units are in kJ/mol. In all scenarios, machine learning models significantly outperform the null model. Standardization and/or centering never improve performance by more than one standard deviation.

Investigated machine learning models: Model names have form *abc*, with *a* indicating the kernel (0 = linear, 1 = squared exponential), *b* indicating standardization (0 = no, 1 = yes), and *c* indicating centering in kernel space (0 = no, 1 = yes). Note that the 011 model is redundant as standardization centers the input vectors.

Name	Description
Ø	Null model
000	Linear kernel, no standardization, no centering
001	Linear kernel, no standardization, centering
010	Linear kernel, standardization, no centering
100	Squared exponential kernel, no standardization, no centering
101	Squared exponential kernel, no standardization, centering
110	Squared exponential kernel, standardization, no centering
111	Squared exponential kernel, standardization, centering

Continued on next page…

Table S2: **Performance of machine learning models.** . . . continued from previous page.

(a) AM1 optimized conformations with DFT-D2 energies.

M.	RMSE	MAE	RMSE %	MAE %	R^2	ΔRMSE	ΔMAE	ΔRMSE %	ΔMAE %	ΔR^2	$\log_2$(nl)	$\log_2$(ls)
Ø	16.79 ± 0.95	12.73 ± 0.44	17.05 ± 1.99	12.98 ± 1.82		23.86 ± 1.35	18.56 ± 0.77	12.75 ± 1.43	9.94 ± 1.28			
000	6.61 ± 1.33	4.09 ± 0.53	6.68 ± 1.36	4.16 ± 0.71	0.85 ± 0.05	9.34 ± 1.87	6.32 ± 0.91	4.97 ± 1.05	3.38 ± 0.61	0.85 ± 0.05	-1.75 ± -3.83	
001	6.63 ± 1.34	4.10 ± 0.52	6.70 ± 1.38	4.17 ± 0.72	0.85 ± 0.06	9.36 ± 1.89	6.33 ± 0.90	4.98 ± 1.06	3.38 ± 0.61	0.85 ± 0.06	-1.70 ± -3.60	
010	6.11 ± 0.99	4.03 ± 0.46	6.18 ± 1.06	4.10 ± 0.66	0.87 ± 0.04	8.62 ± 1.40	6.08 ± 0.75	4.59 ± 0.78	3.25 ± 0.52	0.87 ± 0.04	2.66 ± 0.13	
100	6.55 ± 1.32	4.05 ± 0.52	6.62 ± 1.40	4.12 ± 0.72	0.85 ± 0.05	9.24 ± 1.87	6.24 ± 0.89	4.92 ± 1.06	3.34 ± 0.60	0.85 ± 0.05	-7.94 ± -7.26	7.66 ± 6.98
101	6.45 ± 1.33	4.01 ± 0.51	6.51 ± 1.33	4.08 ± 0.70	0.86 ± 0.05	9.11 ± 1.87	6.17 ± 0.89	4.84 ± 1.01	3.30 ± 0.58	0.86 ± 0.05	-8.01 ± -7.26	7.63 ± 6.79
110	6.12 ± 1.04	3.96 ± 0.47	6.18 ± 1.06	4.03 ± 0.66	0.87 ± 0.04	8.63 ± 1.47	6.01 ± 0.77	4.59 ± 0.77	3.21 ± 0.51	0.87 ± 0.04	-6.38 ± -7.81	8.53 ± 6.80
111	6.08 ± 1.08	3.93 ± 0.47	6.14 ± 1.06	4.00 ± 0.65	0.87 ± 0.04	8.58 ± 1.52	5.96 ± 0.77	4.56 ± 0.77	3.18 ± 0.51	0.87 ± 0.04	-6.40 ± -7.89	8.51 ± 6.34

(b) AM1 optimized conformations with DFT-D3 energies.

M.	RMSE	MAE	RMSE %	MAE %	R^2	ΔRMSE	ΔMAE	ΔRMSE %	ΔMAE %	ΔR^2	$\log_2$(nl)	$\log_2$(ls)
Ø	16.96 ± 0.92	13.44 ± 0.41	19.48 ± 1.77	15.49 ± 1.70		24.09 ± 1.30	18.97 ± 0.74	14.28 ± 1.28	11.27 ± 1.15			
000	5.47 ± 1.05	3.49 ± 0.41	6.29 ± 1.33	4.02 ± 0.67	0.90 ± 0.04	7.73 ± 1.49	5.37 ± 0.72	4.59 ± 0.96	3.19 ± 0.55	0.90 ± 0.04	-1.94 ± -3.83	
001	5.50 ± 1.04	3.50 ± 0.41	6.32 ± 1.33	4.03 ± 0.66	0.90 ± 0.04	7.78 ± 1.48	5.38 ± 0.70	4.61 ± 0.96	3.20 ± 0.55	0.90 ± 0.04	-1.95 ± -3.80	
010	5.00 ± 0.73	3.41 ± 0.34	5.75 ± 1.00	3.93 ± 0.61	0.91 ± 0.02	7.07 ± 1.05	5.13 ± 0.57	4.20 ± 0.72	3.05 ± 0.48	0.91 ± 0.02	2.77 ± 0.24	
100	5.27 ± 1.06	3.39 ± 0.42	6.06 ± 1.32	3.91 ± 0.67	0.91 ± 0.04	7.45 ± 1.50	5.20 ± 0.71	4.42 ± 0.95	3.09 ± 0.55	0.91 ± 0.04	-9.64 ± -10.19	7.79 ± 6.73
101	5.26 ± 1.03	3.39 ± 0.41	6.05 ± 1.31	3.92 ± 0.67	0.91 ± 0.04	7.43 ± 1.45	5.20 ± 0.70	4.41 ± 0.94	3.09 ± 0.55	0.91 ± 0.04	-9.48 ± -9.98	7.73 ± 6.48
110	4.96 ± 0.74	3.34 ± 0.32	5.70 ± 0.99	3.85 ± 0.60	0.92 ± 0.03	7.01 ± 1.05	5.05 ± 0.54	4.16 ± 0.71	3.00 ± 0.47	0.92 ± 0.02	-6.58 ± -7.80	8.52 ± 6.96
111	4.96 ± 0.75	3.34 ± 0.33	5.71 ± 1.02	3.85 ± 0.61	0.92 ± 0.03	7.01 ± 1.07	5.05 ± 0.55	4.16 ± 0.73	3.00 ± 0.48	0.92 ± 0.03	-6.66 ± -7.90	8.66 ± 7.39

(c) DFT-D2 optimized conformations with DFT-D2 energies.

M.	RMSE	MAE	RMSE %	MAE %	R^2	ΔRMSE	ΔMAE	ΔRMSE %	ΔMAE %	ΔR^2	$\log_2$(nl)	$\log_2$(ls)
Ø	20.96 ± 0.85	16.67 ± 0.46	19.70 ± 1.50	15.69 ± 1.37		29.79 ± 1.21	23.72 ± 0.81	14.45 ± 1.06	11.51 ± 0.92			
000	5.72 ± 0.70	3.86 ± 0.37	5.38 ± 0.81	3.64 ± 0.49	0.93 ± 0.02	8.08 ± 0.98	5.86 ± 0.61	3.92 ± 0.60	2.85 ± 0.40	0.93 ± 0.02	-0.54 ± -2.16	
001	5.63 ± 0.65	3.81 ± 0.35	5.30 ± 0.77	3.59 ± 0.47	0.93 ± 0.02	7.95 ± 0.92	5.79 ± 0.58	3.86 ± 0.57	2.81 ± 0.38	0.93 ± 0.02	-0.63 ± -2.38	
010	5.54 ± 1.64	3.67 ± 0.47	5.23 ± 1.74	3.46 ± 0.58	0.93 ± 0.05	7.84 ± 2.33	5.55 ± 0.81	3.82 ± 1.29	2.70 ± 0.49	0.93 ± 0.05	2.39 ± 0.45	
100	5.05 ± 0.61	3.38 ± 0.32	4.75 ± 0.71	3.19 ± 0.44	0.94 ± 0.01	7.13 ± 0.84	5.15 ± 0.51	3.46 ± 0.51	2.51 ± 0.35	0.94 ± 0.01	-6.24 ± -7.64	6.21 ± 4.38
101	5.01 ± 0.59	3.36 ± 0.31	4.72 ± 0.71	3.17 ± 0.44	0.94 ± 0.01	7.07 ± 0.82	5.12 ± 0.50	3.44 ± 0.51	2.49 ± 0.35	0.94 ± 0.01	-6.33 ± -7.38	6.38 ± 4.82
110	4.88 ± 0.66	3.34 ± 0.36	4.60 ± 0.78	3.15 ± 0.47	0.95 ± 0.02	6.90 ± 0.92	5.05 ± 0.58	3.35 ± 0.55	2.46 ± 0.37	0.95 ± 0.02	-6.74 ± -7.75	7.98 ± 5.34
111	5.66 ± 2.77	3.42 ± 0.54	5.37 ± 2.81	3.23 ± 0.63	0.92 ± 0.09	8.00 ± 3.90	5.23 ± 0.97	3.91 ± 2.05	2.55 ± 0.56	0.92 ± 0.09	-6.87 ± -7.95	8.09 ± 5.83

(d) DFT-D2 optimized conformations with DFT-D3 energies.

M.	RMSE	MAE	RMSE %	MAE %	R^2	ΔRMSE	ΔMAE	ΔRMSE %	ΔMAE %	ΔR^2	$\log_2$(nl)	$\log_2$(ls)
Ø	15.06 ± 0.61	11.97 ± 0.32	20.79 ± 1.38	16.55 ± 1.29		21.40 ± 0.87	17.03 ± 0.56	15.24 ± 0.91	12.14 ± 0.80			
000	5.35 ± 0.72	3.51 ± 0.38	7.40 ± 1.17	4.85 ± 0.68	0.88 ± 0.03	7.55 ± 1.02	5.37 ± 0.62	5.39 ± 0.84	3.83 ± 0.55	0.88 ± 0.03	-0.62 ± -2.19	
001	5.22 ± 0.71	3.44 ± 0.38	7.23 ± 1.15	4.77 ± 0.67	0.88 ± 0.03	7.38 ± 1.01	5.27 ± 0.63	5.26 ± 0.83	3.76 ± 0.54	0.88 ± 0.03	-0.69 ± -2.50	
010	5.04 ± 1.62	3.17 ± 0.39	6.99 ± 2.36	4.40 ± 0.72	0.89 ± 0.06	7.13 ± 2.30	4.85 ± 0.69	5.10 ± 1.77	3.47 ± 0.62	0.89 ± 0.06	2.30 ± 0.25	
100	4.99 ± 0.68	3.26 ± 0.36	6.91 ± 1.16	4.51 ± 0.64	0.89 ± 0.03	7.04 ± 0.96	5.00 ± 0.60	5.03 ± 0.82	3.57 ± 0.52	0.89 ± 0.03	-6.30 ± -7.28	6.50 ± 5.81
101	4.97 ± 0.66	3.25 ± 0.35	6.89 ± 1.14	4.50 ± 0.64	0.89 ± 0.03	7.02 ± 0.93	4.98 ± 0.58	5.01 ± 0.81	3.56 ± 0.51	0.89 ± 0.03	-6.20 ± -7.33	6.49 ± 5.92
110	4.45 ± 0.62	2.97 ± 0.33	6.16 ± 1.10	4.12 ± 0.63	0.91 ± 0.03	6.28 ± 0.87	4.53 ± 0.54	4.49 ± 0.77	3.24 ± 0.50	0.91 ± 0.03	-6.64 ± -7.79	8.35 ± 6.00
111	4.48 ± 0.63	2.99 ± 0.33	6.21 ± 1.12	4.14 ± 0.63	0.91 ± 0.03	6.32 ± 0.89	4.55 ± 0.54	4.52 ± 0.79	3.25 ± 0.50	0.91 ± 0.03	-6.63 ± -7.91	8.42 ± 6.53
